# Assessment of transfer methods for comparative genomics of regulatory networks in bacteria

**DOI:** 10.1186/s12859-016-1113-7

**Published:** 2016-08-31

**Authors:** Sefa Kılıç, Ivan Erill

**Affiliations:** Department of Biological Sciences, University of Maryland Baltimore County (UMBC), Baltimore, MD 21250 USA

**Keywords:** Comparative genomics, Regulatory networks, Transcription factor, Benchmark, Transfer, Motifs, Site search, Regulon, AUC

## Abstract

**Background:**

Comparative genomics can leverage the vast amount of available genomic sequences to reconstruct and analyze transcriptional regulatory networks in Bacteria, but the efficacy of this approach hinges on the ability to transfer regulatory network information from reference species to the genomes under analysis. Several methods have been proposed to transfer regulatory information between bacterial species, but the paucity and distributed nature of experimental information on bacterial transcriptional networks have prevented their systematic evaluation.

**Results:**

We report the compilation of a large catalog of transcription factor-binding sites across Bacteria and its use to systematically benchmark proposed transfer methods across pairs of bacterial species. We evaluate motif- and accuracy-based metrics to assess the results of regulatory network transfer and we identify the precision-recall area-under-the-curve as the best metric for this purpose due to the large class-imbalanced nature of the problem. Methods assuming conservation of the transcription factor-binding motif (motif-based) are shown to substantially outperform those assuming conservation of regulon composition (network-based), even though their efficiency can decrease sharply with increasing phylogenetic distance. Variations of the basic motif-based transfer method do not yield significant improvements in transfer accuracy. Our results indicate that detection of a large enough number of regulated orthologs is critical for network-based transfer methods, but that relaxing orthology requirements does not improve results. Using the transcriptional regulators LexA and Fur as case examples, we also show how DNA-binding domain sequence similarity can yield confounding results as an indicator of transfer efficiency for motif-based methods.

**Conclusions:**

Counter to standard practice, our evaluation of metrics to assess the efficiency of methods for regulatory network information transfer reveals that the area under precision-recall (PR) curves is a more precise and informative metric than that of receiver-operating-characteristic (ROC) curves, confirming similar findings in other class-imbalanced settings. Our systematic assessment of transfer methods reveals that simple approaches to both motif- and network-based transfer of regulatory information provide equal or better results than more elaborate methods. We also show that there are not effective predictors of transfer efficacy, substantiating the long-standing practice of manual curation in comparative genomics analyses.

**Electronic supplementary material:**

The online version of this article (doi:10.1186/s12859-016-1113-7) contains supplementary material, which is available to authorized users.

## Background

The availability of complete genome sequences for related organisms can be effectively leveraged to study transcriptional regulatory networks (TRN) [1]. In the past decade, comparative genomics approaches have been routinely employed to study bacterial transcriptional regulatory networks, or regulons, controlled by a single transcription factor (TF). These studies have enabled the identification of core network elements and niche-specific adaptations, providing insights into the evolution of these systems [2–7]. A conventional TRN comparative genomics analysis typically involves three well defined steps [1, 8, 9]. The first step consists in the transfer of available information on the regulatory network (i.e. known TF-binding sites and/or regulated genes) to the species under analysis, in order to infer the TF-binding motif in these target species. The second step involves a genome-wide search for putative TF-binding sites in the target genomes using the inferred TF-binding motifs. In the third step, search results from multiple genomes are integrated across orthologs, based on the assumption that only orthologs of regulated genes will systematically display TF-binding sites in their promoter regions.

The power of comparative genomics arises from the aggregation of predictions in multiple genomes under the assumption of functional selection, which dramatically reduces the number of false positives [8]. However, the effectiveness of this approach depends crucially on the success of the initial transfer step. Information from a reference TRN can be transferred though the assumption of a conserved TF-binding motif, a conserved regulon, or a combination of the two [1, 6]. As a consequence, several methods have been proposed to transfer regulatory information across species. The simplest approach, here called “direct transfer”, is to use the reference TF-binding motif to search for sites in the target genome. The target motif is hence implicitly defined as a subset of the highest scoring sites in that genome [2–4, 7]. In “direct discovery”, the direct transfer scheme is further elaborated by applying a motif discovery or optimization algorithm on a set of high-scoring sites from the target genome [9, 10]. The alternative to TF-binding motif-based transfer is to assume conservation of gene content in the TRN. Regulon or “network transfer” is typically implemented through the detection of orthologs for genes in known regulated operons. The promoter regions of the corresponding operons in target genomes are then analyzed with a motif discovery tool to elicit the TF-binding motif [11–14]. If network information is not available, a minimal network consisting only of the TF-coding gene can be postulated under the assumption of self-regulation, and the TF-binding motif can be inferred with motif discovery tools applied to promoter region of the TF-coding gene [15–17]. Lastly, motif- and network-based transfer approaches can be combined in “mixed transfer” to minimize false positives, at the expense of lowered sensitivity [6, 9, 18].

The limited availability of experimental data on TF-binding sites has hindered attempts at systematically assessing and comparing methods for transfer of transcriptional regulatory networks. Early studies on TRN transfer indicated that the efficiency of methods based on TF-binding motif transfer faced a sharp drop-off with increasing sequence divergence among the TF orthologs [18]. Later studies exposed the limitations of network-based transfer methods, due to shortcomings in orthology detection methods and the flexible nature of bacterial TRNs [19–21]. It has been suggested that mixed transfer methods provide better results [6], but the scarcity and distributed nature of TF-binding site data have to date prevented systematic benchmarking of these methods. In this work, presented at the 2015 International Symposium on Bioinformatics Research and Applications [22], we report the mining and integration of experimental TF-binding site data from multiple databases into a unified catalog. Leveraging this resource, we performed a systematic evaluation of TRN transfer methods across pairs of species and using multiple metrics. In agreement with previous reports, our results reveal that motif-based transfer methods perform best, but decay sharply at high TF sequence divergence. In contrast, the efficiency of network-based transfer methods is poor and weakly dependent of phylogenetic distance, while mixed methods do not significantly improve the results of motif-based transfer methods. Our analysis also highlights the inadequacy of receiver-operating-characteristic (ROC) curves in heavily unbalanced settings and indicates that the precision-recall (PR) area-under-the-curve (AUC) is the most informative statistic for assessment of transfer results. We evaluate predictive measures for transfer accuracy and discuss their applicability in the context of comparative genomics analyses.

## Results and discussion

### Data compilation and evaluation of metrics for the assessment of transfer methods

To perform a systematic analysis of methods for the transfer of transcriptional regulatory networks in Bacteria, we compiled data from five major databases reporting experimentally-validated TF-binding sites across the Bacteria domain. After consolidating replicates, we obtained a catalog of 7,603 TF-binding sites for 344 TFs in 166 species (Additional file 1). To analyze TRN transfer, we focused on TF/species pairs that contained at least 10 binding sites for the same TF in both species. The resulting dataset contains 179 TF-specific species pairs for 15 different TFs across 35 bacterial species and is dominated by instances of the global transcriptional regulators LexA and Fur (Additional file 2).

**Fig. 1:**
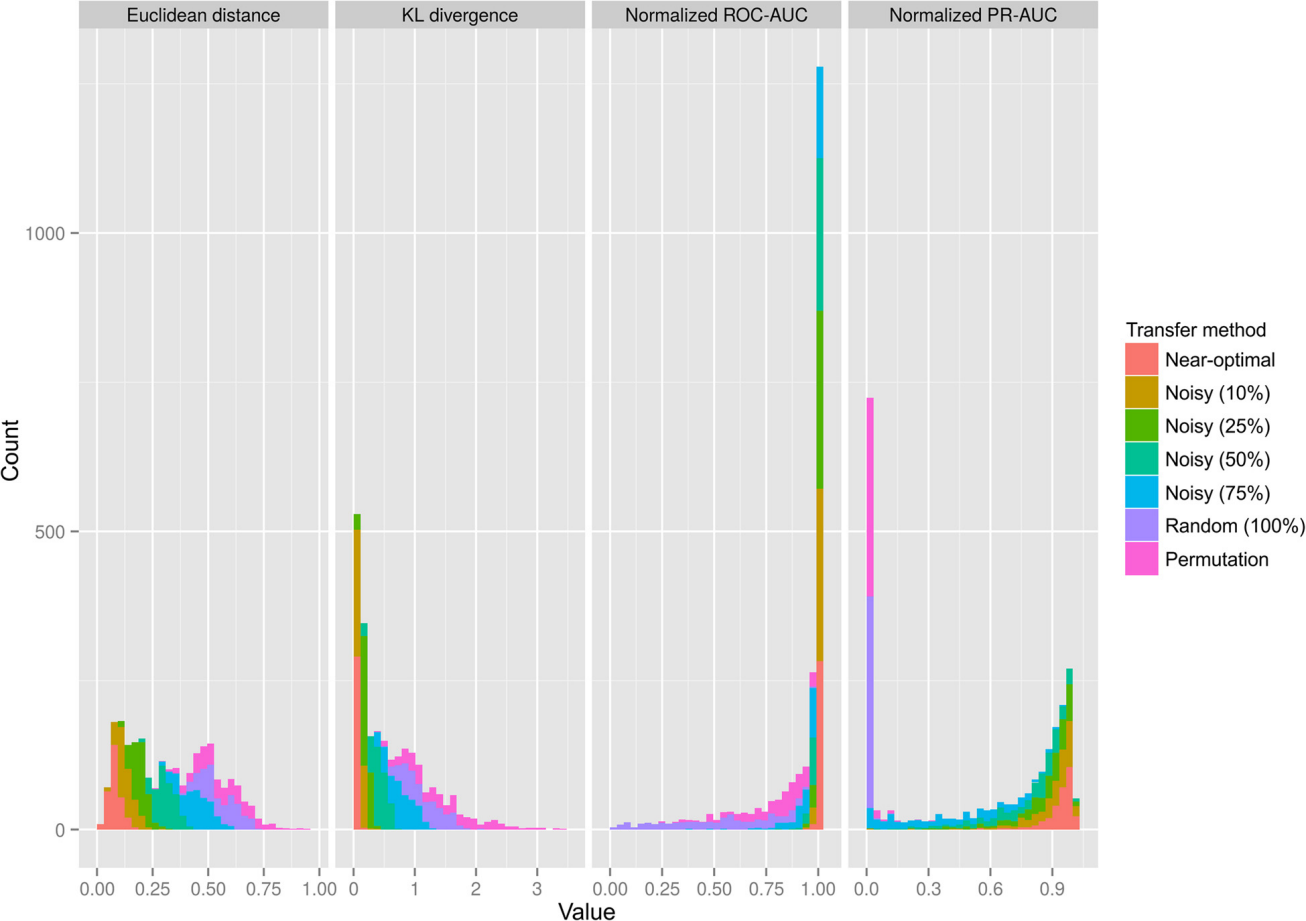
Comparison of methods for the evaluation of TRN transfer. The plots show the histogram of Euclidean distance, KL divergence, ROC-AUC and PR-AUC values for simulated bidirectional transfers between 179 TF-specific species pairs using different degrees of noise (10, 25, 50 and 75 % of random sites sampled when creating pseudo-replicates), as well as their random and permuted controls. The y-axis shows the number of transfers within each binning value on the x-axis. ROC-AUC and PR-AUC values are normalized to the respective AUC of the known target TF-binding motif to compensate for the decreased search efficiency of low information content motifs

The establishment of an adequate metric is a necessary and crucial element in a benchmark study. When transferring TRNs from a reference species to a target species for comparative genomics, the result of the transfer process is an inferred TF-binding motif in the target species. Given the inferred and known TF-binding motifs in the target genome, one can evaluate the accuracy of the transfer process by directly comparing the motifs or by assessing the efficiency of the inferred motif at retrieving the known TF-binding sites in a genome search. Here we focused on the Euclidean distance and the Kullback–Leibler (KL) divergence as well-established motif comparison functions based on the position-specific frequency matrix (PSFM) defined by the motif [23], and on two standard metrics for classification accuracy based on the area-under-the curve (AUC) derived from a TF-binding site search process: the receiver-operating-characteristic (ROC) AUC and the precision-recall (PR) AUC [24, 25]. To assess the efficacy of each method at discriminating the efficacy of TRN transfer, we simulated transfers by defining inferred motifs as noisy pseudo-replicates or permutations of the known collection of binding sites in the target genomes (Additional file 3). We then assessed the quality of these simulated transfers against the known target motif using the four metrics outlined above (Fig. 1).

As it can be seen in Fig. 1, both the Euclidean distance and KL-divergence perform only moderately well at discriminating the results of simulated noisy transfers (containing 50 % and 75 % random sites) from completely random or permuted motifs. This result is partly due to the fact that the expectation for random motifs is not to yield maximum distance, narrowing the useful range of motif comparison metrics. The two other contributing factors are the high-dimensionality of TF-binding motifs, which is known to decrease the relative contrast of L-norms [26], and the presence of low information bearing positions in most TF-binding motifs. Low information positions are intrinsically close in PSFM space, artificially increasing the similarity between motifs for both metrics [27]. As a result, in both cases, random and permuted motifs display a considerable spread, leading to significant overlap with the results obtained for simulated noisy transfers. In practice, transfer methods frequently generate motifs comparable to the noisy transfers simulated here, and their overlap with random controls therefore complicates the interpretation of transfer results.

Accuracy metrics based on a genome-wide search for known TF-binding sites should in principle provide a more informative metric of the effectiveness of the transfer process, since they evaluate the ability of the inferred motif to locate true binding sites in the target genome. In contrast with motif comparison methods, the expectation for accuracy metrics is hence that incorrect or random transfers should yield very low AUC values. However, this does not happen for the ROC-AUC, a widely adopted metric in bioinformatics [28]. This result is due to the large class imbalance in the TF-binding search problem, where a handful of known true sites must be distinguished from the genome background. Even though ROC curves scale properly with class imbalance [29], they are ill-suited to discriminate between classifiers in a heavily imbalanced context, because the negative class dominates the computation of the ROC-AUC [30]. The net result of this effect is a compression of AUC scores for noisy motifs into a very narrow range (0.9–1.0), making discrimination between near-optimal and noisy transfers almost impossible. This compression affects also the results obtained for random and permuted motifs, which spread all the way up to 0.95 AUC scores, further complicating the interpretation of transfer results. By focusing on the ratio between true and false positives (precision) and otherwise ignoring the negative class, the PR-AUC generates scores are not compressed by class imbalance [28, 30]. As it can be seen in Fig. 1, the PR-AUC effectively exploits its range to discriminate between noisy transfers and systematically assigns very low values to random and permuted motifs. Hence, the PR-AUC provides the most effective metric for the benchmarking of TRN transfer methods and was used in all subsequent analyses reported here.

### Comparison of transfer methods

Motif-based and network-based transfer methods rely on different assumptions about the evolutionary dynamics of transcriptional regulatory networks. The former assume that the TF-binding motif is conserved to some extent, while the latter assume that the gene components of the regulon are conserved. As a result, it is presumed that motif-based methods will perform poorly at large phylogenetic distances due to expected divergence in the TF-binding motif, whereas network-based methods are expected to be more resilient to phylogenetic distance if the biological function of the regulatory network is preserved. Interestingly, there is evidence supporting and invalidating both assumptions and their corollaries. The SOS response transcriptional regulator LexA, for instance, has been shown to target widely diverging motifs in relatively close species [31], whereas some transcriptional regulators, like the heat-shock response repressor HrcA or the arginine repressor ArgR, are known to preserve their binding motif across Bacteria to different extents [3, 32]. On the other hand, regulon composition has been documented to vary significantly even among closely related species [4–6, 21]. Furthermore, CRP/FNR-type regulators have been shown to control completely different networks using closely related motifs across Bacteria [33, 34].

Here we tested the robustness of TRN transfer methods by evaluating the PR-AUC of inferred TF-binding motifs in 179 TF-specific species pairs, using three motif-based and three network-based methods, as well as a combination of motif- and network-based methods (Additional file 4). The motif-based transfer methods include direct transfer and direct discovery methods. In *direct transfer*, the reference collection of TF-binding sites is used directly to determine the inferred collection by searching promoter regions in the target genome. In *direct discovery*, the results of a relaxed search and their surrounding regions are used as input for a motif discovery algorithm, with the goal of generating a motif better adapted to the target genome. The network-based transfer methods evaluated differ in how they map genes regulated in the reference genome to the target genome. This mapping can be based on the detection of direct orthology for genes in regulated operons, their functional assignment using Clusters of Orthologous Groups (COGs) or orthology detection using their interacting network partners. The mixed approach combines a relaxed TF-binding motif search with the restriction that identified sites must be associated with genes mapped with any of the three network-based transfer approaches.

**Fig. 2:**
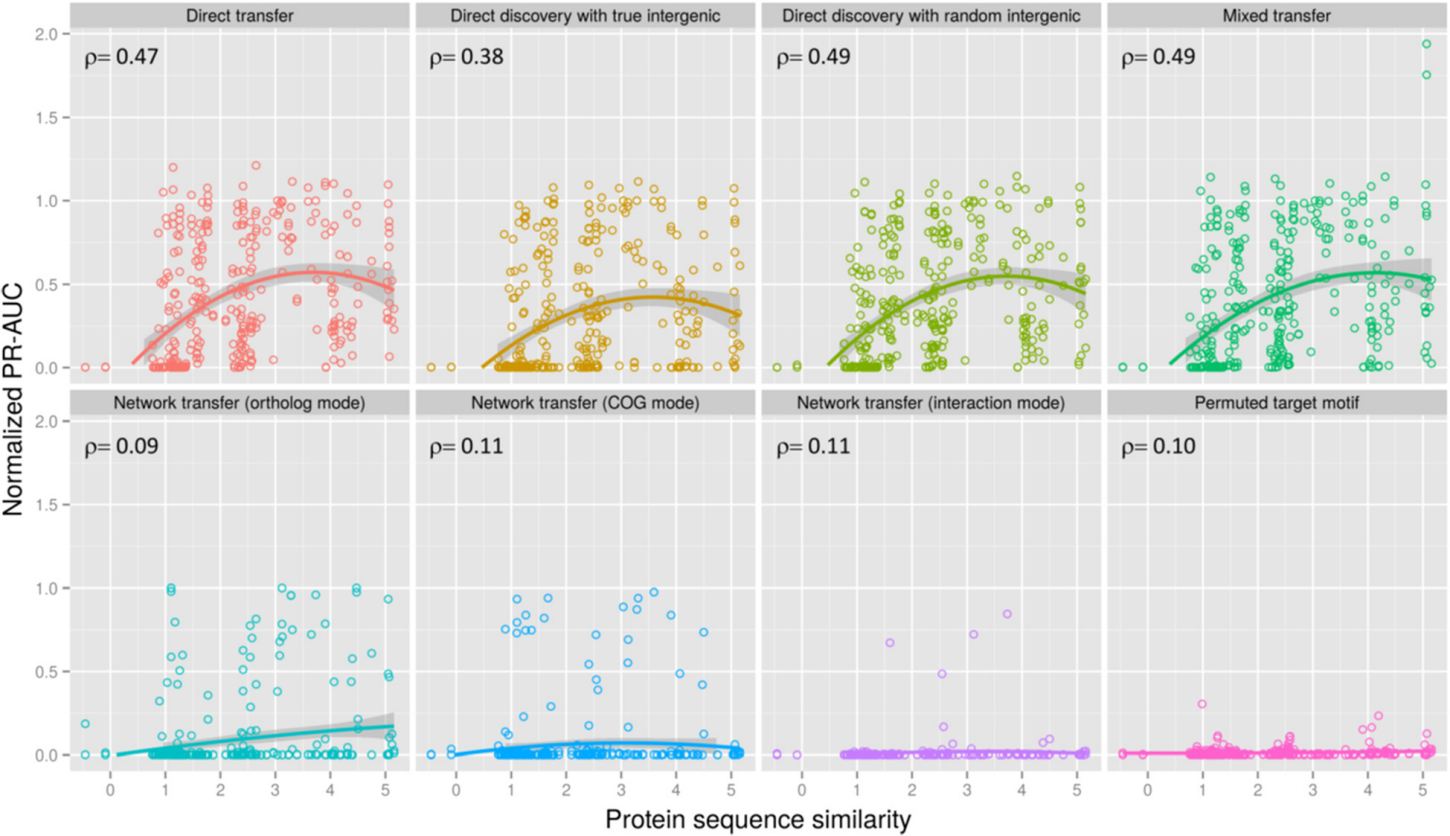
Comparison of TRN transfer methods. The plots show the PR-AUC of bidirectional transfers between 179 TF-specific species pairs using motif-based (*direct transfer*, *direct discovery* with true intergenic and *direct discovery* with random intergenic) and transfer-based methods (*ortholog*, *COG* and *interaction* modes), as well as a mixed model integrating *direct transfer* and the union of all network-based transfer methods. The results obtained with a permutation of the target motif are shown for comparison. The x-axis denotes protein similarity as the BLOSUM62 score of the ungapped pair-wise alignment between reference and target TF protein sequences. PR-AUC values are normalized to the respective AUC of the known target TF-binding motif to compensate for the decreased search efficiency of low information content motifs. Spearman correlation coefficients (*ρ*) of PR-AUC with protein similarity are shown for each case

In agreement with previous research, the results shown in Fig. 2 reveal that the effectiveness of motif-based transfer methods declines rapidly with decreasing sequence similarity between the TF protein sequences [18]. In contrast, the results of network-based transfer methods show only moderate correlation with protein sequence similarity, but these methods perform poorly when compared to motif-based transfer methods. Among the three mapping modes analyzed for network-based transfer, the direct *ortholog* mode provides the best results, but is still only able to generate successful transfers in 15 % of the cases. The poor efficiency of network-based transfer methods supports previous research highlighting the evolutionary flexibility of bacterial transcriptional regulatory networks, which decreases their expected overlap in gene composition [5, 19–21]. The low efficiency of network-based transfers could therefore stem from an inability of these transfer methods to identify conserved regulated genes (low recall) or from the inclusion of too many orthologs without conserved regulation in the transfer process (low precision). The interplay between these factors should explain the significant differences observed between network transfer modes, since these variants are intended to progressively relax the concept of orthology in order to enhance recall. To analyze their relative contributions, we computed the Spearman correlation coefficient between the search PR-AUC reported in Fig. 2 and the precision/recall of the network transfer process for the different transfer modes. We find that recall (*ρ*_*r*_ = 0.115), rather than precision (*ρ*_*p*_ = 0.106), is the dominant factor for the more restrictive *ortholog* mode. This indicates that detecting enough orthologs with conserved regulation is critical for proper motif inference. However, the situation is reversed for the more relaxed *COG* (*ρ*_*r*_ = 0.089; *ρ*_*p*_ =0.191) and *interaction* (*ρ*_*r*_ = -0.108; *ρ*_*p*_ =0.140) modes. These results suggest that the increase in mapped orthologs that are not regulated in the target genome (loss of precision) overcomes any substantial enhancement in recall achieved by relaxed mapping modes (Additional file 5).

In contrast with network-based transfer methods, the different implementations of motif-based transfer yield very similar results (Fig. 2). Using the reference motif to search promoter regions and define the putative target motif (*direct transfer*) provides results comparable to those obtained with other motif-based transfer methods and robust with respect to the specific threshold used to define the motif (Additional file 6). The use of MEME in *direct discovery* transfers to rediscover the TF-binding motif, which has been postulated to refine and better adapt the inferred motif to the target genome [10], does not provide significant improvements over *direct transfer*. In fact, when performing motif discovery on the promoter region surrounding the identified sites, MEME may identify other genomic elements (e.g. Pribnow boxes) as the best motifs, decreasing the accuracy of the method. Performing motif inference on the identified sites surrounded by random promoter regions prevents this effect, but does not yield a systematic improvement in PR-AUC values over *direct transfer*. Finally, the *mixed mode* approach, which has been associated with enhanced specificity [6, 9], did not yield a systematic improvement over *direct transfer* either.

### Predictive indicators of transfer accuracy

Our comparative analysis of transfer methods (Fig. 2) indicates that, even at relatively close phylogenetic distances, both motif- and network-based transfer methods may provide inaccurate results. Hence, manual curation of transfer results, which has been the *de facto* standard for comparative genomics of TRN in Bacteria [2, 4, 8, 35], appears to be a necessary requisite to ensure the reliability of any subsequent comparative genomics analyses. Leveraging the TF-binding site catalog compiled here, we attempted to identify predictive indicators of transfer accuracy for motif- and network-based transfer methods. Several studies have exploited sequence similarity in the DNA-binding domain of the TF as a criterion for clustering putative regulatory regions in motif discovery [15, 17, 33, 36]. The rationale for this approach is that similar DNA-binding domains will target conserved TF-binding motifs. Hence, it is plausible to assume that DNA-binding domain sequence similarity could be an efficient predictor of transfer accuracy for motif-based transfer methods.

**Fig. 3:**
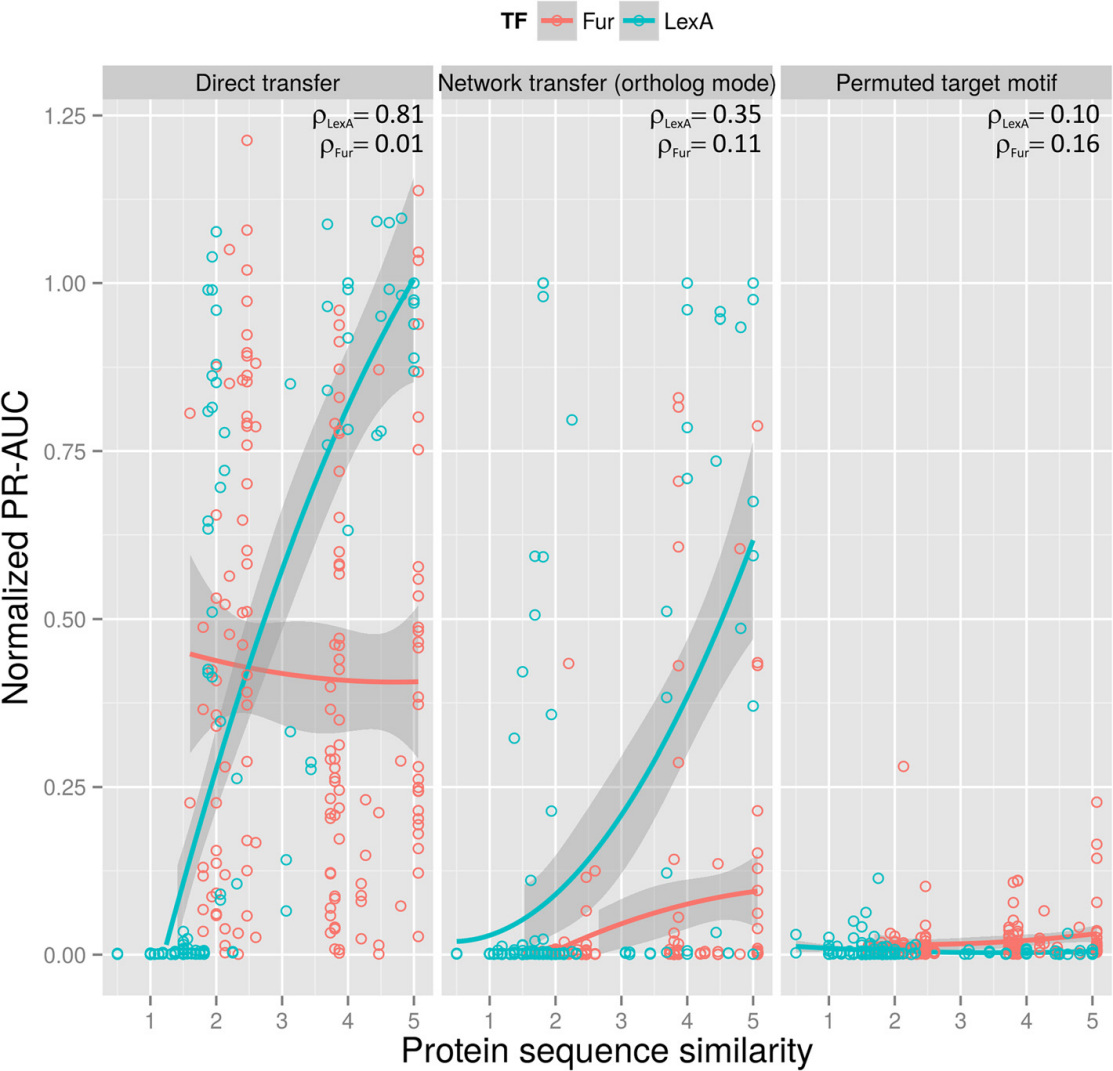
Assessment of protein sequence similarity as a predictor of TRN transfer method accuracy. The plots show the PR-AUC of bidirectional transfers between 143 (66 LexA, 77 Fur) TF-specific species pairs using motif-based (*direct transfer*) and transfer-based methods (*ortholog mode*) as a function of TF protein sequence similarity. The results obtained with a permutation of the target motif are shown for comparison. The x-axis denotes protein similarity as the BLOSUM62 score of the ungapped pair-wise alignment between reference and target TF protein sequences. PR-AUC values are normalized to the respective AUC of the known target TF-binding motif to compensate for the decreased search efficiency of low information content motifs. Spearman correlation coefficients (*ρ*) of PR-AUC with protein similarity are shown for each case

To test whether DNA-binding domain sequence similarity is a good predictor of transfer accuracy, we examined transfer accuracy for two transcription factors (LexA and Fur) on which we had abundant TF-binding site data and for which the DNA-binding domain has been experimentally determined [37, 38]. The results shown in Fig. 3 reveal that DNA-binding domain sequence similarity is not a universal predictor of transfer accuracy. For LexA, DNA-binding domain sequence similarity shows a clear correlation (Spearman *ρ* = 0.81) with motif-based transfer accuracy, but this correlation is completely absent for Fur (*ρ* = 0.01). Our results therefore suggest that for transcription factors (like Fur) targeting a conserved binding motif, the efficiency of motif-based methods will not significantly decrease with sequence divergence in the DNA-binding domain. In contrast, and in agreement with previous findings [18], the accuracy of motif-transfer methods is expected to decrease sharply for LexA and other transcription factors that have significantly altered their binding specificity through evolution. In this context, DNA-binding domain sequence similarity provides a more accurate indicator of transfer efficiency than phylogeny (Additional file 7).

**Fig. 4:**
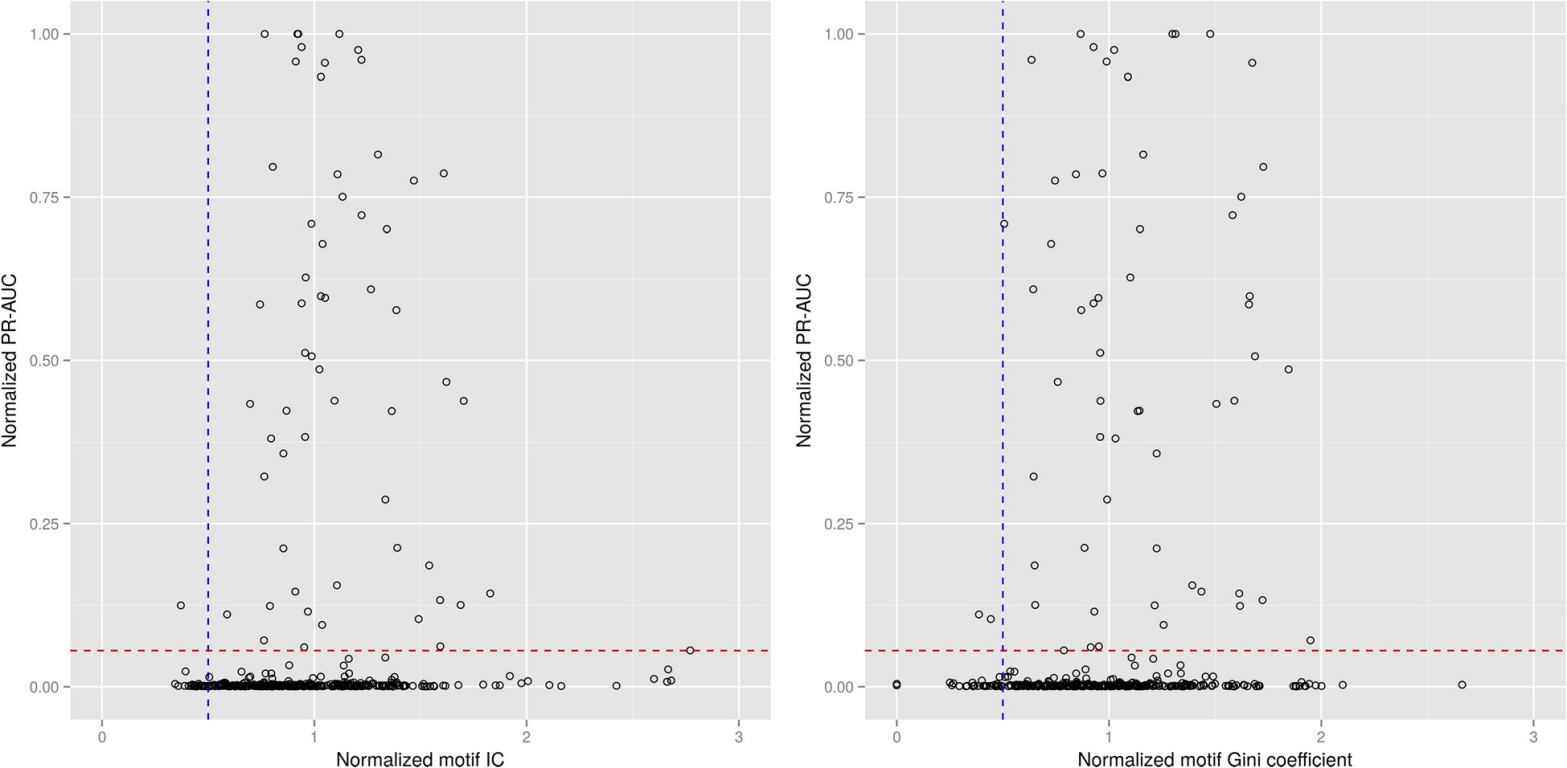
Assessment of IC (left) and Gini coefficient (right) as predictive indices of accuracy for network-based transfer methods. The plots show the distribution of PR-AUC from bidirectional transfers between 179 TF-specific species pairs using network transfer (*ortholog mode*), with respect to each index. PR-AUC values are normalized to the respective AUC of the known target TF-binding motif to compensate for the decreased search efficiency of low information content motifs. IC and Gini coefficient values are normalized to those observed on the known reference motif. The dotted vertical line designates 0.5 normalized motif IC/Gini. The dotted horizontal line identifies the PR-AUC value (0.056) two standard deviations above the mean obtained from simulated transfers with permuted motifs.

The results shown in Fig. 3 also indicate that DNA-binding domain sequence similarity correlates weakly with accuracy for network-based transfer methods. DNA-binding domain sequence similarity is a proxy for phylogenetic distance (Additional file 7), and the observed loss of accuracy of network-based transfer methods is hence congruent with decreased overlap in the components of regulatory networks for increasing phylogenetic distances [5, 6, 21]. It is possible, however, to conceive of other measures that might function as predictive indices of transfer accuracy for network-based transfer methods. These methods rely on motif discovery algorithms, like MEME, to infer the functional motif for the transcription factor in the target species, providing some theoretical bounds on expected properties of the inferred motifs. For instance, the information content (IC) of a TF-binding motif is known to correlate with the number of operons regulated by the TF [39]. Hence, if the size of the regulatory network is assumed to remain relatively constant, we expect the IC of the inferred TF-binding motif to be similar to that observed in the reference species. In a similar vein, the distribution of information in a TF-binding motif is related to the structure of the TF and its mode of binding (e.g. homodimers typically target palindromic motifs) [17, 32, 40]. It follows that measures of information distribution in inferred TF-binding motifs, such as the Gini coefficient [41], should not differ much between reference and inferred motifs under the assumption of conserved protein structure. We analyzed the predictive power of these indices on PR-AUC using the complete TF-binding site catalog (Fig. 4). While neither index can reliably identify successful transfers, both indices reveal clear cutoff values beyond which accurate transfers should not be expected. For both IC and Gini coefficient, a relative index of 0.5 with respect to the known reference motif is a strong indicator of unsuccessful transfer (96 % and 94 % unsuccessful transfers for IC and Gini relative values below 0.5, compared to 83 % for both IC and Gini values above 0.5), and the evidence suggests that this may also be true for IC and Gini values above 2.

## Conclusions

Transferring known information about transcriptional regulatory networks from reference to target species is a critical step in comparative genomics analyses. In this work, we compiled a catalog of known TF-binding sites in Bacteria and performed a methodic evaluation of assessment metrics in order to perform the first systematic analysis of different transfer methods. Our results identify the precision-recall area-under-the-curve as the most reliable metric for transfer efficiency. We also show that motif-based transfer methods dramatically outperform network-based approaches, but their efficiency may decrease sharply with increasing phylogenetic distance. We evaluate some predictive indicators of transfer accuracy and show that they are not consistent or precise enough to enable full automation of TRN transfer. Our results hence support the long-standing practice of manual curation in comparative genomics analyses and reveal that the introduction of more elaborate methods does not clearly benefit motif- or network-based transfer approaches.

## Methods

### TF-binding site and genome data

Experimentally-validated TF-binding sites were compiled from CollecTF [42], a Bacteria domain-wide TF-binding site database, and four model-organism databases: RegulonDB, CoryneRegNet, DBTBS and MtbRegList [43–46]. Data from these databases was downloaded and merged after removal of duplicates and of data without supporting experimental evidence. To evaluate transfer methods across pairs of species, only regulons with at least 10 experimentally-validated TF-binding sites for a given TF in both species were used. Complete genome sequences and annotations for species with available TF-binding site data were downloaded from the NCBI RefSeq database [47]. Operon predictions for all genomes and COG annotations for protein-coding genes were obtained from the DOOR database [48, 49].

### TF-binding site search and motif discovery

For TF-binding site search, only the regions spanning from −300 bp to +50 bp relative to the corresponding gene translation start site were considered. Site search was implemented using custom scripts based on standard Biopython library functions [50]. The searched regions were scanned with a sliding window, evaluating each position with a position-specific scoring matrix (PSSM) based on a uniform background mononucleotide model [51]. Motif discovery on selected sequences was performed with MEME, using command line settings *-zoops -revcomp -dna*, and maximum and minimum motif widths, respectively, of 150 % and 50 % of the reference motif width [52].

### Transfer methods

Two main motif-based transfer methods were implemented. In *direct transfer*, a position-specific scoring matrix (PSSM) is built from the reference collection of binding sites and used to scan the promoter regions of the genome of interest to identify putative sites [51]. Under the assumption that regulon size is conserved to a first approximation, the target motif is defined as composed of the highest scoring *N*_*T*_ sites. *N*_*T*_ *= α* · *N*_*R*_ *· G*_*T*_*/G*_*R*_, where *N*_*R*_ is the number of sites in the reference species, *G*_*T*_ and *G*_*R*_ are genome lengths for target and reference species, respectively, and *α* is used as a scaling factor to modulate specificity (*α* = 1.25). *Direct discovery* uses a relaxed scaling factor *α* (*α* = 2.50) to generate a larger collection of putative sites. These sites and their adjoining intergenic regions are used to rediscover the TF-binding motif with MEME in *direct discovery with true intergenic*. For *direct discovery with random intergenic*, the genomic intergenic regions are substituted by 100 bp stretches randomly generated following the intergenic region nucleotide frequencies.

Network-based transfer was implemented using three different criteria to map regulated genes in the reference genome to target genomes. In *ortholog mode*, orthologs of all genes belonging to regulated operons were detected as best reciprocal BLAST hits between species pairs using a minimum e-value threshold of 10^−10^ [53]. In *COG mode*, all genes in the target genome mapping to the same COG as genes in regulated operons of the reference genome were considered functional orthologs. In *interaction mode*, direct interacting partners for regulated genes in the reference genome were identified using the STRING database [54]. The orthologs of these genes on target genomes were detected through reciprocal BLAST and used to define putative regulatory networks. In all three cases, the promoters of all target operons containing mapped genes were then used for motif discovery with MEME.

*Mixed transfer* uses a relaxed (*α* = 2.50) TF-binding site search with the reference TF-binding motif to define a set of putative sites in the target genome. This collection of putative sites is filtered by retaining only those sites next to operons containing genes that have been mapped to the target genome by any of the network-based transfer methods.

### Assessment metrics

Two main types of assessment metrics were used to gauge the efficacy of TF motif-based transfer methods: motif comparison and performance metrics. Motif comparison methods involve the direct comparison of the TF-binding motifs inferred by the transfer method and the motif generated from the known collection of regulated genes in the target genome. To compare motifs of different lengths, the two collections of binding sites are shifted with respect to each other to obtain the gapless alignment that maximizes the information content (IC) of the joint collection [39]. The aligned region of each collection is then used to compute its position-specific frequency matrix (PSFM). The similarity between the two PSFM is evaluated using either the Euclidean distance or the Kullback–Leibler (KL) divergence of the inferred motif from the known target motif [23]. Performance metrics evaluate the ability of the inferred motif to retrieve the known target sites when searching an equal number of promoter regions from the target genome containing, and not containing, known sites. To assess true positives, the maximum IC alignment between target and inferred collections is used to compute the offset between predicted and known sites. The accuracy of the search using the inferred TF-binding motif is then evaluated as the area-under-the-curve (AUC) of the receiver-operator-characteristic (ROC) or precision-recall (PR) curve, computed with the scikit-learn Python library functions [24, 25].

Control experiments for TRN transfer were generated in two different ways. Noisy transfers were simulated by defining the inferred motif as a mixture pseudoreplicate of the known target collection and sequences from the promoter region of the target genome. Given a mixture weight *ω*, the pseudoreplicate is obtained by sampling, with replacement, (1-*ω*) · *N* sites from the known target collection and *ω* · *N* sequences of length *L* from the promoter region of the target genome (where *N* and *L* are, respectively, the number and width of sites in the known target collection) (Additional file 3). Noisy transfers were generated for 0.1, 0.25, 0.5 and 0.75 values of *ω*, simulating increasingly inefficient transfers. Permuted transfers were obtained by randomly sampling, without replacement, the columns of the known target motif. A transfer was considered successful if its PR-AUC was larger than two standard deviations above the mean PR-AUC value observed for permuted transfers.

## Abbreviations

AUC, area under the curve; BLAST, basic local alignment search tool; COG, clusters of orthologous groups; CRP, camp receptor protein; FNR, fumarate and nitrate reduction protein; IC, information content; KL, kullback–leibler; MEME, multiple EM for motif elicitation; PR, precision-recall; PSFM, position specific frequency matrix; PSSM, position specific scoring matrix; ROC, receiver-operating-characteristic; TF, transcription factor; TRN, transcriptional regulatory network

## Additional files

Additional file 1: Distribution of TF-binding sites in the compiled catalog by originating database. (DOCX 11 kb) Additional file 2: Distribution by TF of the number of species pairs with at least 10 experimentally validated TF-binding sites in each species. (DOCX 11 kb) Additional file 3: Generation of noisy transfers and controls. (PPT 136 kb) Additional file 4: Schematic representation of transfer methods. (PPT 197 kb) Additional file 5: Precision-Recall plot for different network transfer modes. (PNG 201 kb) Additional file 6: Direct transfer results for different thresholds. (PNG 599 kb) Additional file 7: Efficiency of transfer methods by phylogeny. (PNG 150 kb)
