# Supplementary material for: Assessment of transfer methods for comparative genomics of regulatory networks in bacteria
Source: BMC Bioinformatics. 2016 Aug 31;17(Suppl 8):277. doi: 10.1186/s12859-016-1113-7 (PMC5009822; doi:10.1186/s12859-016-1113-7)
Supplement: Additional file 1: — Distribution of TF-binding sites in the compiled catalog by originating database. (DOCX 11 kb) [file 12859_2016_1113_MOESM1_ESM.docx]

**Additional file 1:** Distribution of TF-binding sites in the compiled catalog by originating database.

| **Database** | **Number of binding sites** |
| --- | --- |
| CollecTF | 4,942 |
| DBTBS | 116 |
| MtbRegList | 202 |
| CoryneRegNet | 196 |
| RegulonDB | 2,147 |
