# Supplementary material for: Assessment of transfer methods for comparative genomics of regulatory networks in bacteria
Source: BMC Bioinformatics. 2016 Aug 31;17(Suppl 8):277. doi: 10.1186/s12859-016-1113-7 (PMC5009822; doi:10.1186/s12859-016-1113-7)
Supplement: Additional file 2: — Distribution by TF of the number of species pairs with at least 10 experimentally validated TF-binding sites in each species. (DOCX 11 kb) [file 12859_2016_1113_MOESM2_ESM.docx]

**Additional file 2:** Distribution by TF of the number of species pairs with at least 10 experimentally validated TF-binding sites in each species.

| **Transcription factor** | **Number of species pairs** |
| --- | --- |
| Fur | 154 |
| LexA | 134 |
| CcpA | 20 |
| PhoP | 12 |
| CodY | 12 |
| OmpR | 6 |
| CRP | 4 |
| RpoN | 4 |
| FNR | 2 |
| PurR | 2 |
| DtxR | 2 |
| ArgR | 2 |
| PvdS | 2 |
| CsgD | 2 |
