# Supplementary material for: Assessment of transfer methods for comparative genomics of regulatory networks in bacteria
Source: BMC Bioinformatics. 2016 Aug 31;17(Suppl 8):277. doi: 10.1186/s12859-016-1113-7 (PMC5009822; doi:10.1186/s12859-016-1113-7)
Supplement: Additional file 3: — Generation of noisy transfers and controls. (PPT 136 kb) [file 12859_2016_1113_MOESM3_ESM.ppt]

## Slide 1
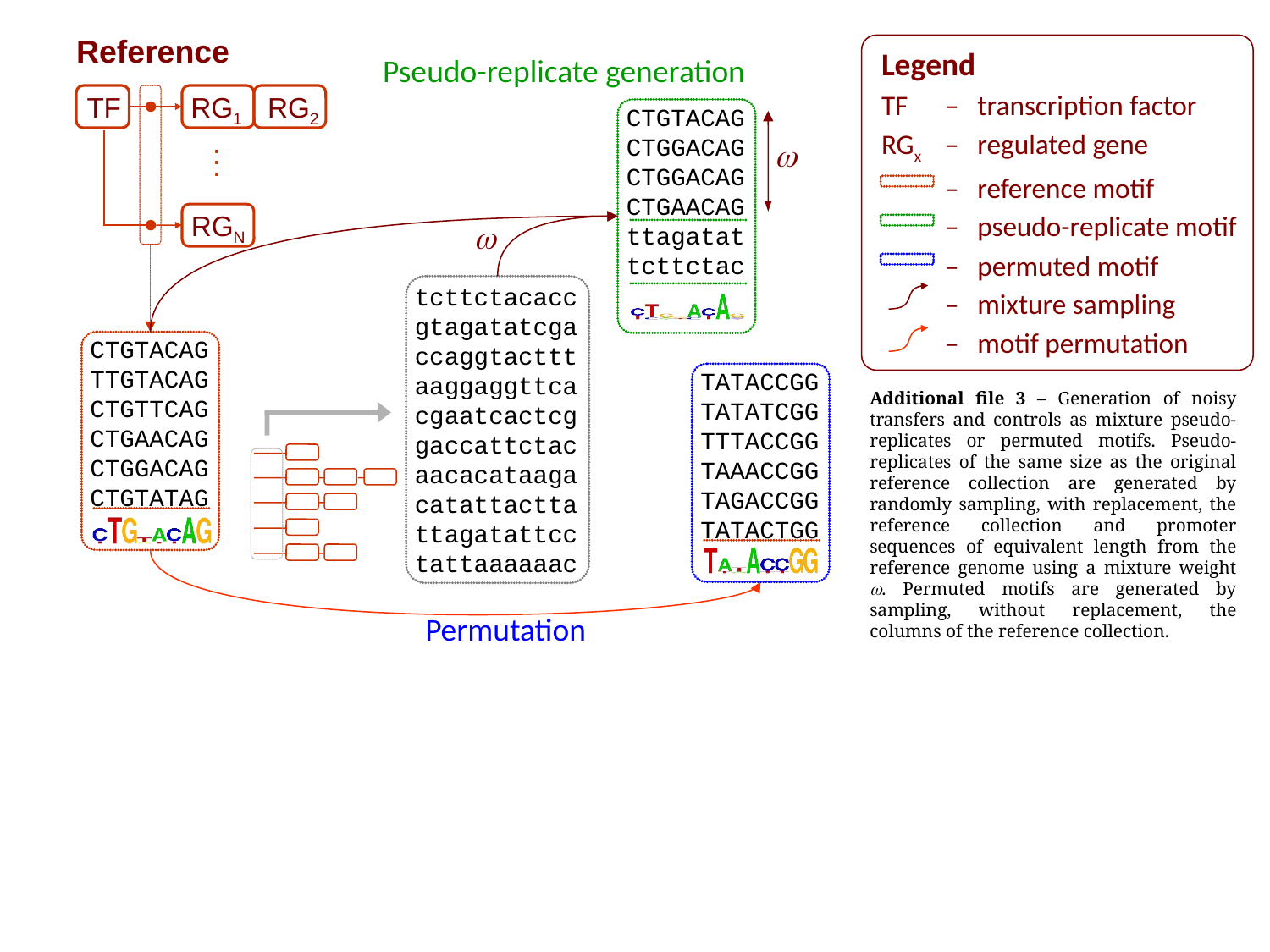

Reference
Legend
TF	–	transcription factor
RGx	–	regulated gene
	–	reference motif
	–	pseudo-replicate motif
	–	permuted motif
	–	mixture sampling
	–	motif permutation
Pseudo-replicate generation
TF
RG1
RG2
CTGTACAG
CTGGACAG
CTGGACAG
CTGAACAG
ttagatat
tcttctac

.
.
.
RGN

tcttctacacc
gtagatatcga
ccaggtacttt
aaggaggttca
cgaatcactcg
gaccattctac
aacacataaga
catattactta
ttagatattcc
tattaaaaaac
CTGTACAG
TTGTACAG
CTGTTCAG
CTGAACAG
CTGGACAG
CTGTATAG
TATACCGG
TATATCGG
TTTACCGG
TAAACCGG
TAGACCGG
TATACTGG
Additional file 3 – Generation of noisy transfers and controls as mixture pseudo-replicates or permuted motifs. Pseudo-replicates of the same size as the original reference collection are generated by randomly sampling, with replacement, the reference collection and promoter sequences of equivalent length from the reference genome using a mixture weight . Permuted motifs are generated by sampling, without replacement, the columns of the reference collection.
Permutation
