# Supplementary material for: Assessment of transfer methods for comparative genomics of regulatory networks in bacteria
Source: BMC Bioinformatics. 2016 Aug 31;17(Suppl 8):277. doi: 10.1186/s12859-016-1113-7 (PMC5009822; doi:10.1186/s12859-016-1113-7)
Supplement: Additional file 4: — Schematic representation of transfer methods. (PPT 197 kb) [file 12859_2016_1113_MOESM4_ESM.ppt]

## Slide 1
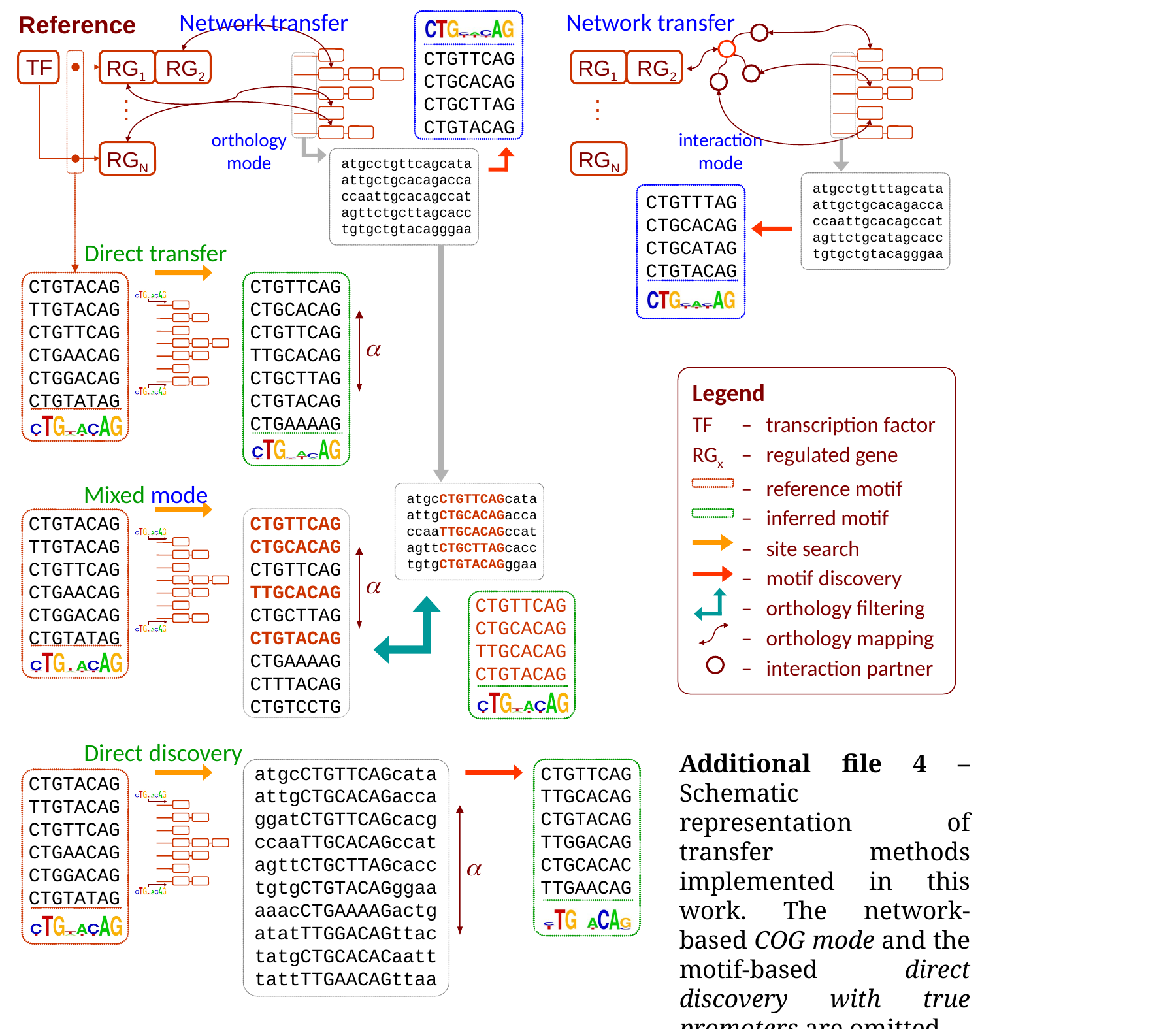

Network transfer
Network transfer
Reference
CTGTTCAG
CTGCACAG
CTGCTTAG
CTGTACAG
TF
RG1
RG2
RG1
RG2
.
.
.
.
.
.
orthology mode
interaction mode
RGN
RGN
atgcctgttcagcata
attgctgcacagacca
ccaattgcacagccat
agttctgcttagcacc
tgtgctgtacagggaa
atgcctgtttagcata
attgctgcacagacca
ccaattgcacagccat
agttctgcatagcacc
tgtgctgtacagggaa
CTGTTTAG
CTGCACAG
CTGCATAG
CTGTACAG
Direct transfer
CTGTACAG
TTGTACAG
CTGTTCAG
CTGAACAG
CTGGACAG
CTGTATAG
CTGTTCAG
CTGCACAG
CTGTTCAG
TTGCACAG
CTGCTTAG
CTGTACAG
CTGAAAAG
α
Legend
TF	–	transcription factor
RGx	–	regulated gene
	–	reference motif
	–	inferred motif
	–	site search
	–	motif discovery
	–	orthology filtering
	–	orthology mapping
	–	interaction partner
Mixed mode
atgcCTGTTCAGcata
attgCTGCACAGacca
ccaaTTGCACAGccat
agttCTGCTTAGcacc
tgtgCTGTACAGggaa
CTGTACAG
TTGTACAG
CTGTTCAG
CTGAACAG
CTGGACAG
CTGTATAG
CTGTTCAG
CTGCACAG
CTGTTCAG
TTGCACAG
CTGCTTAG
CTGTACAG
CTGAAAAG
CTTTACAG
CTGTCCTG
α
CTGTTCAG
CTGCACAG
TTGCACAG
CTGTACAG
Direct discovery
Additional file 4 – Schematic representation of transfer methods implemented in this work. The network-based COG mode and the motif-based direct discovery with true promoters are omitted.
atgcCTGTTCAGcata
attgCTGCACAGacca
ggatCTGTTCAGcacg
ccaaTTGCACAGccat
agttCTGCTTAGcacc
tgtgCTGTACAGggaa
aaacCTGAAAAGactg
atatTTGGACAGttac
tatgCTGCACACaatt
tattTTGAACAGttaa
CTGTTCAG
TTGCACAG
CTGTACAG
TTGGACAG
CTGCACAC
TTGAACAG
CTGTACAG
TTGTACAG
CTGTTCAG
CTGAACAG
CTGGACAG
CTGTATAG
α
