# Supplementary figures and images for: Assessment of transfer methods for comparative genomics of regulatory networks in bacteria
Source: BMC Bioinformatics. 2016 Aug 31;17(Suppl 8):277. doi: 10.1186/s12859-016-1113-7 (PMC5009822; doi:10.1186/s12859-016-1113-7)

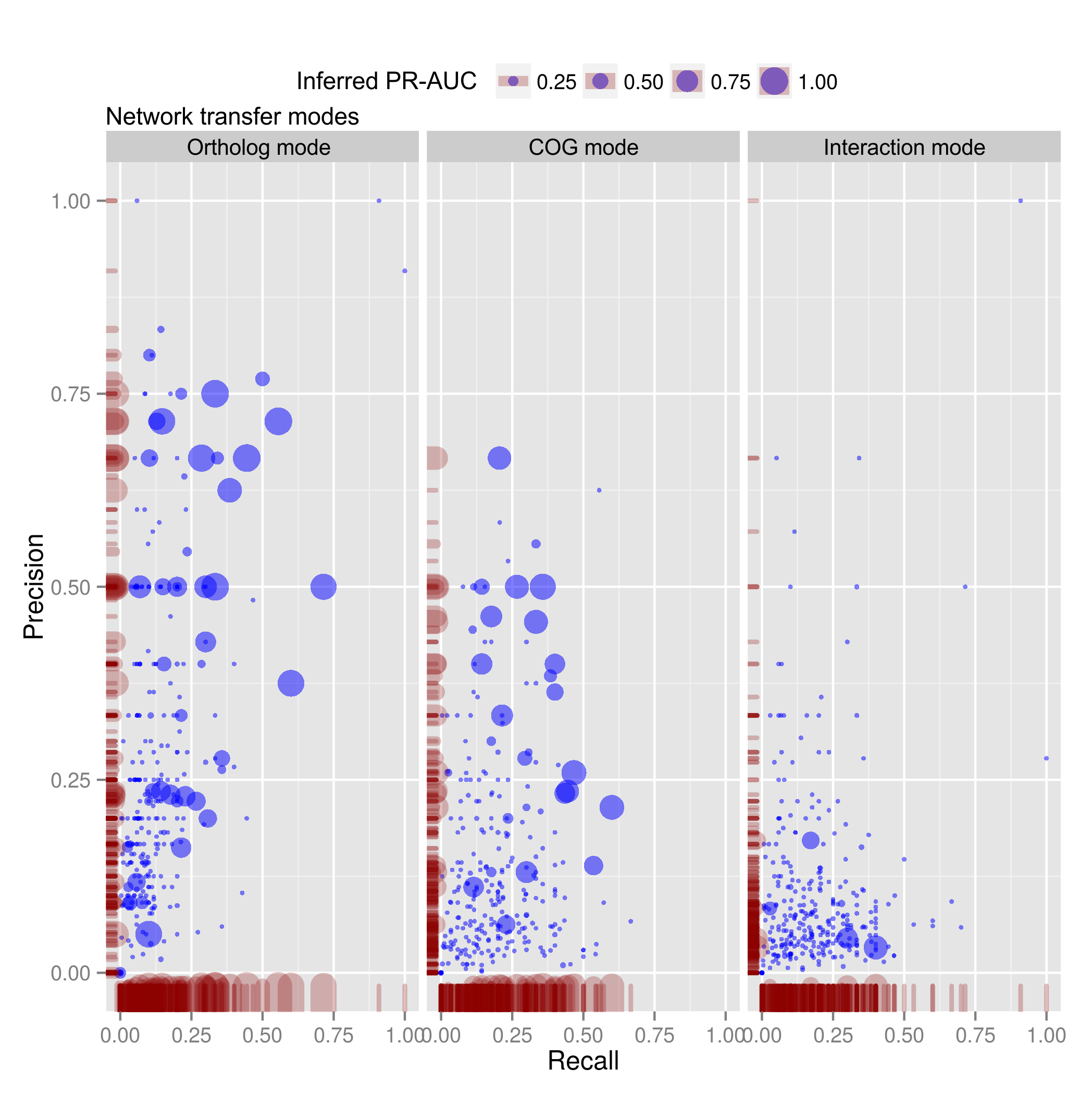

Supplement: Additional file 5: — Precision-Recall plot for different network transfer modes. (PNG 201 kb) [file 12859_2016_1113_MOESM5_ESM.png]

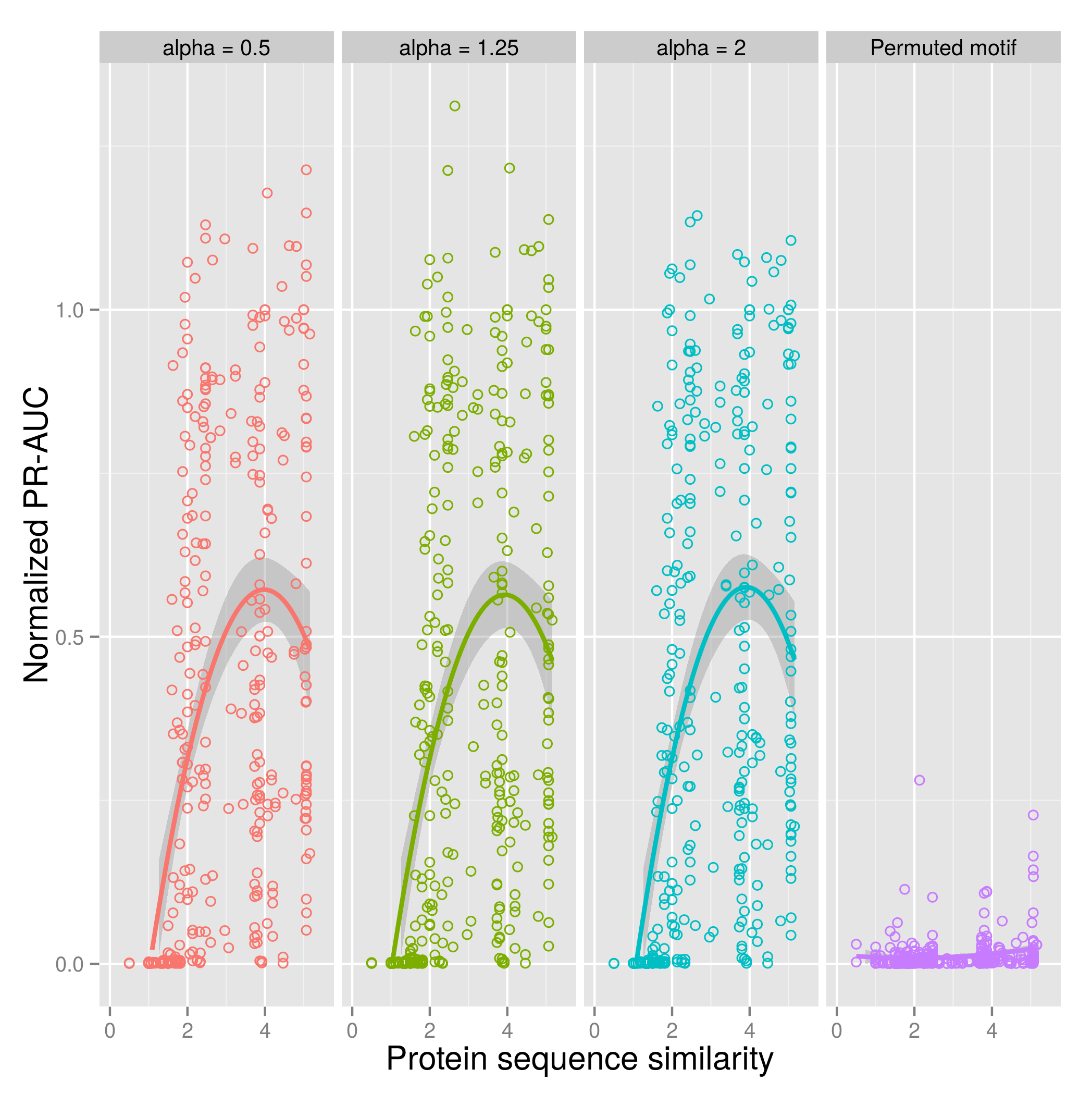

Supplement: Additional file 6: — Direct transfer results for different thresholds. (PNG 599 kb) [file 12859_2016_1113_MOESM6_ESM.png]

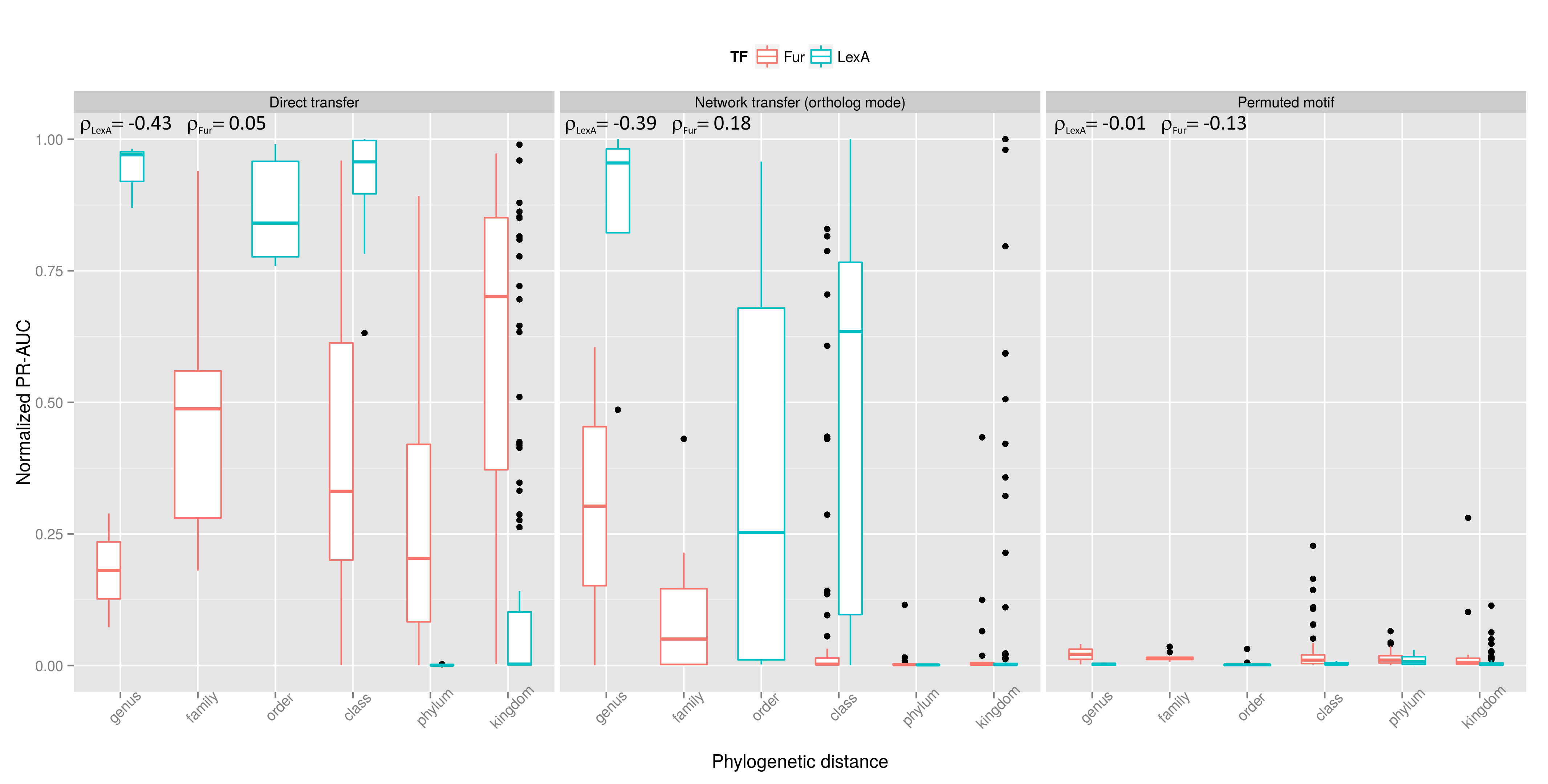

Supplement: Additional file 7: — Efficiency of transfer methods by phylogeny. (PNG 150 kb) [file 12859_2016_1113_MOESM7_ESM.png]
